# Joining of Dissimilar Al and Mg Metal Alloys by Friction Stir Welding

**DOI:** 10.3390/ma15175901

**Published:** 2022-08-26

**Authors:** Ramandeep Singh Sidhu, Raman Kumar, Ranvijay Kumar, Pankaj Goel, Sehijpal Singh, Danil Yurievich Pimenov, Khaled Giasin, Krzysztof Adamczuk

**Affiliations:** 1 Department of Mechanical and Production Engineering, Guru Nanak Dev Engineering College, Ludhiana 141006, Punjab, India; 2 Department of Mechanical Engineering, University Center for Research and Development, Chandigarh University, Ludhiana 140413, Punjab, India; 3 Department of Business Management, Guru Nanak Institute of Management and Technology, Ludhiana 141008, Punjab, India; 4 Department of Automated Mechanical Engineering, South Ural State University, Lenin Prosp. 76, 454080 Chelyabinsk, Russia; 5 School of Mechanical and Design Engineering, University of Portsmouth, Portsmouth PO1 3DJ, UK; 6 Faculty of Mechanical Engineering, University of Zielona Gora, 4 Prof. Z. Szafrana Street, 65-516 Zielona Gora, Poland

**Keywords:** friction stir welding (FSW), aluminum alloy, magnesium alloy, dissimilar joints, cost analysis, mechanical properties, microstructure, fractography, potentiodynamic corrosion test

## Abstract

In engineering applications, such as automobile, marine, aerospace, and railway, lightweight alloys of aluminum (Al) and magnesium (Mg) ensure design fitness for fuel economy, better efficiency, and overall cost reduction. Friction stir welding (FSW) for joining dissimilar materials has been considered better than the conventional fusion welding process because of metallurgical concerns. In this study, dissimilar joints were made between the AA6061 (A), AZ31B (B), and AZ91D (C) combinations based on the varying advancing side (AS) and retreating side (RS). The dissimilar joints prepared by the FSW process were further characterized by tensile testing, impact testing, corrosion testing, fracture, and statistical and cost analysis. The results revealed a maximum tensile strength of 192.39 MPa in AZ91 and AZ31B, maximum yield strength of 134.38 MPa in a combination of AA6061 and AZ91, maximum hardness of 114 Hv in AA6061 and AZ31B, and lowest corrosion rate of 7.03 mV/A in AA6061 and AZ31B. The results of the properties were supported by photomicrographic fracture analysis by scanning electron microscopy (SEM) observations. Further, the performance of dissimilar joints was statistically analyzed and prioritized for preference by similarity to the ideal solution (TOPSIS) method.

## 1. Introduction

Welding is a process of permanently joining different materials, and welding of similar and dissimilar Al and Mg alloys has become a significant research hotspot [1,2]. Nowadays, the main thrust is replacing dense alloys with lightweight alloys with FSW for weight reduction and fuel conservation in automobiles, making electric tools, marine equipment, space shuttles, turbines, and agricultural machinery for high-strength joints [3]. FSW is a solid-state welding operation that results in better mechanical and microstructural properties than conventional techniques [4]. Al of density 2.7 g/cm^3^ and its alloys are considered lightweight alloys, and are commonly available and widely used for packing films, power devices, electronic devices, automobile appliances, wind-power control, and solar power controllers [5]. AA series Al alloys have exceptional properties, for example, being lightweight, high strength, highly conductive in nature, antierosion, and environment friendly. Mg of density 1.74 g/cm^3^ is another abundant element found in seawater and is lighter than Al [6]. Therefore, Mg is an element used in the aerospace, automobile, and shipbuilding industries [7]. Mg alloys have decent properties, such as specific strength, sound-damping ability, formability, and reusability [8]. On the other hand, Mg has less hardness, strength, fatigue, stiffness, [9] creep, and corrosion-resistance properties at room temperature due to the HCP crystal structure [10].

There is much scope for improvement in research on FSW. An AZ31 Mg alloy was butt welded by FSW. Different tool profiles of H13 tool steel and welding parameters were used for welding, and defects of joints were analyzed [11]. AZ31-Mg alloy temperature details of FSW and processing were described using the parametric modeling approach. The outcome was beneficial for simulating weld temperature for processing diverse metals and alloys [12]. Two plates of Mg alloy AZ91 of 6 mm thick were joined with FSW at a WS of 28 to 56 mm/min., and tool-rotation speed ranged from 710 to 1400 rpm. Tensile, fluorescent penetrant, and optical microscopy tests were conducted to observe defects. Sound joints were attained using a threaded straight cylindrical pin profile with a shoulder diameter of 18 mm, rotational speed (R_s) of 710 rpm, and a WS of 28 mm/min. The tapered cylindrical pin profile was unsuitable for FSW of Mg alloy AZ91 [13]. Mg (AZ31B) and Al (AA6061) alloys had a plate thickness of 6 mm joined by FSW. Joints equipped with 21 mm shoulder diameter displayed greater tensile properties and efficiency (89%) than lower-strength BM [14]. Mg AZ31B-H24 and AA6061-T6 Al were joined with FSW, with a pin range varying from 3.25 mm and 3.75 mm and welding speed (WS) 600–1000 rpm. Tunnel defects were produced at WS 4.25 mm pin at 800 and 1000 rpm [15]. It is troublesome when intermetallic phases occur during dissimilar welding using conventional welding, because—depending on the thickness—they can seriously compromise the joint integrity [16]. AZ31 and AZ91 Mg alloy sheets were joined at 1400 rpm and 25 mm/min. of feed with FSW. Hardness has shown an increasing trend because of SZ grain refinement, the existence of Mg_17_Al_12_ particles, and solid solution strengthening in the SZ [17]. The impact of changing the feed rate while maintaining a fixed rotational speed on the mechanical and microstructural properties of FSW AA5754 was studied. FSW resulted in dynamic recrystallization, which resulted in microstructural alterations in various zones [18]. The more significant research conducted while joining AA6061, AZ31B and AZ91 with FSW is shown in Table 1. It shows the workpiece, tool geometry and material, welding parameters and properties analyzed, and remarks.

Table 1 shows that AA6061, AZ31B and AZ91 were successfully welded utilizing FSW. Mechanical properties like hardness, tensile, and impact energy were studied frequently, as well as microstructure and macrostructure. However, significantly less research was conducted on potentiodynamic corrosion of AA6061, AZ31B, and AZ91 materials. Some significant studies are presented in Table 2. This shows a need to study the potentiodynamic corrosion of selected AA6061, AZ31B, and AZ91 materials’ dissimilar FSW joints.

The literature reveals that very few studies have been reported correlating mechanical and thermal properties with corrosion, fracture, and cost. Also, the importance of this research is in the cost analysis, statistical analysis, and detailed multiobjective optimization strategy for selecting process parameters that have been less reported. In this study, tensile testing, impact testing, corrosion testing, fracture, and statistical and cost analysis of the joints of Al and Mg alloys manufactured by the FSW process were conducted. This review does not cover a combined effect of all properties of FSW. The TOPSIS technique prioritizes dissimilar friction stir welded joints.

## 2. Materials and Methods

We adopted an experimental research design to analyze the mechanical properties of FSW butt joints of dissimilar Al and Mg metal alloys by friction stir welding. We took sampling units of the dissimilar metals aluminum (Al) AA6061 (A), magnesium (Mg), and AZ31B (B) and AZ91D (C) combinations. Their selection was due to their excellent mechanical properties, corrosion resistance, and castability. The universal milling machine model DMU 50T of Deckel Maho manufracturer made in Bielefeld, Germany, was used for FSW. A workpiece of rectangular shape of 300 × 55 × 6 mm^3^ was utilized. The dissimilar joints prepared by the FSW process were characterized by tensile testing, impact testing, corrosion testing, fracture testing, and statistical and cost analysis. Photomicrographic fracture analysis by SEM observations supported the results. The measured output parameters wee hardness, corrosion, tensile strength, yield strength, impact, and elongation. Pearson’s correlation coefficient, linear regression analysis, coefficient of determination, and ANOVA statistical techniques were used to analyze the available data. The statistical software SPSS version 26 of JMP statistical manufacturer, Miami, FL, USA and Microsoft Excel of Microsoft Global Services Centre (Bangalore, India) processed the raw information.

In the present work, FSW of dissimilar metals of Al and Mg were performed in the Central Control Room, Focal Point, Ludhiana, Punjab, India. The details of workpiece material, tool material, equipment used, FSW parameters, and responses are presented in this section. Mg AZ31 and AZ91 were purchased from NUFIT Piping Solution, Mumbai, Maharashtra, India, and Al AA6061 was from Ludhiana, Punjab, India. The contents of the materials and the composition symbols used are given in Table 3.

**Figure 1:**
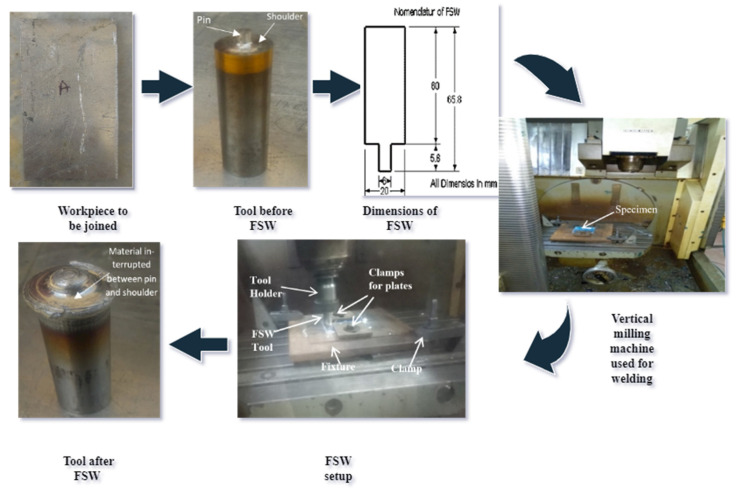
FSW methodology: workpiece, tool used, FSW tooling, tool after joining.

The methodology is shown in Figure 1, and it provides information about the tool set up for FSW and the FSW H13 tool. A nonconsumable straight cylindrical shoulder pin tool was used, with a shoulder diameter of 20 mm, shoulder length of 60 mm, pin diameter of 6 mm, pin length of 5.8 mm, and total tool length of 65.8 mm. The tool utilized before and after welding is shown in Figure 1.

**Figure 2:**
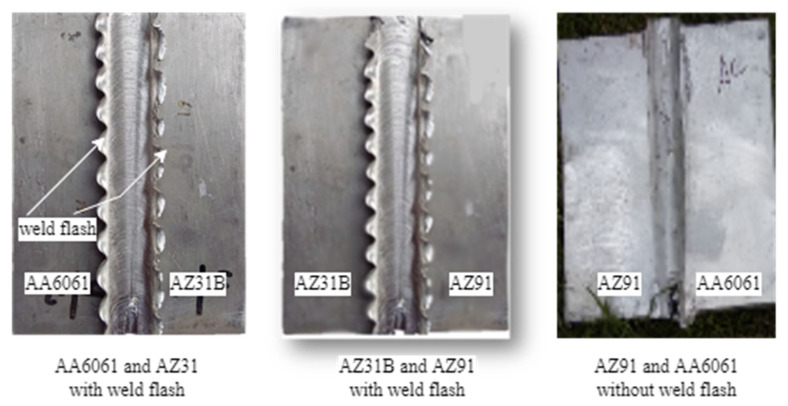
FSW joint view with or without flash.

A butt joint was welded by FSW of similar and dissimilar metals of Al and Mg. The joints were welded at changed R_s (1000, 1200, 1400, 1500 rpm) and WS (12, 20, 24 m/min.) of the tool. The feed rate was 40 mm/min constant, and 12 trials were performed. The best defect-free results of the FSW joint were obtained at an R_s of 1200 rpm and WS of 24 m/min. The experimental design of dissimilar alloys consisted of Al alloy AA6061 (A), Mg alloy AZ31B (B), and AZ91 (C). FSW was performed with AA6061 and AZ31B, AZ31 and AA6061, AA6061 and AZ91, AZ91 and AA6061, AZ31B and AZ91, and AZ91 and AZ31B, whereas AA6061 and AZ31B (AB) consisted of alloy A on the RS, and B was on the AS. After designing the experiments, the workpiece of A, B, and C alloys of rectangular shape 300 × 55 × 6 mm^3^ was prepared. FSW was executed on the vertical milling machine, and clamps were used to fix fixtures at the milling machine bed surface. The fixture consisted of a backplate, clamps, and nut bolt. A view of the welded joints is given in Figure 2. The WS was set at 24 m/min and R_s 1200 rpm as per the experimental design. All tests on combinations of designs for FSW were repeated three times. The workpieces were cleaned with acetone after performing FSW. Finally, the specimens were prepared to perform different destructive and nondestructive tests. The destructive tests included tensile, impact, hardness, and corrosion tests. All the observations of the destructive tests were noted three times, and further analysis was conducted on the means. The nondestructive test included the Microstructure of FS welded specimens.

The fractography test was performed on the fractured workpiece from the tensile test. Finally, an SEM test was used to check the cross-section breakage of tensile specimens. Samples were prepared of size 50 × 16 × 6 mm^3^, with a 45° V notch of 2 mm depth. The pendulum was adjusted at a 135° angle at a certain height. For the tensile test, the specimen dimensions were considered as per American Society Test and Material (ASTM), West Conshohocken, PA, USA ASTM-E8-Type IV specifications, with length of 100 mm, thickness of 6 mm, and width of 10 mm.

The corrosion test was conducted while considering chloride ion concentration in 1 mol/L of pH value 11 and 60 min of exposure time. Six samples were ground with silicon carbide papers from 100 up to 3000 grit, then polished using a polishing cloth in the presence of a diamond polisher (GEM industries manufacturer, Rajasthan, India) up to a surface roughness of 0.5 μm to see the microstructure of BM, HAZ, TMAZ, and SZ and microhardness. An etched solution was prepared with 2 g NaOH and 100 mL distilled water to clean the welded butt joints, and they were also washed with 5 mL HNO_3_ in 95 mL distilled water. Cotton swabs were used to apply etchant to Mg-Al samples and water to remove etchant, and tissue paper to remove water from the surface of samples. Etchant was applied for microhardness and SEM specimens.

### 2.1. Microhardness, Tensile, and Impact Tests

Hardness tests measure the resistance of a material to indentation. Microhardness refers to the process of measuring the hardness of materials using small applied loads. Hardness tests were performed on Vickers Hardness Testers (Model: VM-50) made in India. The test was conducted as per ASTM-E8-Type IV specification standards. A diamond indenter was used, pressed on the workpiece’s surface with a 100 gm load up to 10 s. Afterwards, the indentation was measured, and the material’s hardness had an inverse relation to the indentation area, as shown in Equation (1), *d*_1_ and *d*_2_ are the diagonal lengths of the indentor [23].

(1) $$VHN=1.72\times \frac{F}{d_{2}d_{1}}$$

Tensile tests were performed on an Instron 5500 R electromechanical universal testing machine made in the United Kingdom, and the experiment was conducted as per ASTM-E8-Type IV specifications. There are two types of impact tests—Charpy and Izod. The Charpy test and experiment were conducted to determine ductile and brittle fracture. The facility is available at GNDEC Ludhiana, Punjab, India. All tests were performed at room temperature.

### 2.2. Microstructure, Fractography, and Corrosion Tests

A low-magnification optical microscope (Light Optical Compound Biological, Changzhou, China) was used to identify the microstructure of FSW specimens. Tests were performed in Ludhiana, Punjab, India. Optical photomicrographs were taken at ×100 magnification to check the microstructures of base materials and materials in AS and RS, HAZ, SZ, and TMAZ of both AS and RS sides. In addition, analysis was conducted to check the mechanical deformation due to the involvement of heat at each phase.

A fractography test was performed using a series scanning electron microscope (model JSM-6510, Japan). Used tensile specimens were used as fractography specimens. The test was performed at Thapar University, Patiala, Punjab, India. It used magnification of 5 to × 300,000 (on 128 mm × 96 mm image size), and the electrical image shifted up to ±50 μm (WD = 10 mm) and accelerating voltage up to 0.5 kV to 30 kV.

The polarization tests were performed in corrosion cells with NaCl solution. Each corrosion cell consisted of a reference electrode, cathode, and counter electrode. Silver (Ag) was used as the reference electrode, the specimen as cathode, and titanium (Ti) as the counter electrode. The NaCl solution was applied to specimens by dipping specimens into the solution. A rate of 1 mV/s was carried out for the polarization scan. The corrosion rate η was calculated in millimeters/year (mm/a) using Equation (2).

(2) $$\eta =\frac{J_{corr}\times F}{1000}$$

The current density $J_{corr}$ was measured in a/m^2^ using Equation (3):

(3) $$J_{corr}=\frac{b_{a}\times b_{c}}{\left[2.3R_{p}\;\left(b_{a}+b_{c}\right)\right]}$$

where *b_a_* is represented as anodic Tafel slope in V, *b_c_* is represented as cathodic Tafel slope in V, and *R_p_* is the polarization resistance in Ω/m^2^. Finally, the metal factor *F* was calculated using Equation (4):

(4) $$F=t\times \frac{k}{\rho }$$

where *t* is the seconds in a year, *ρ* is the density in g/cm^3^, and *k* is the electrochemical equivalent in g/C. Corrosion rate is a function of chloride ion concentration (*c*), pH value (*P*), and exposure time (*t*), the units of corrsion rate is mm/(a mm year) and a is area of sample in mm^2^ [33].

## 3. Results and Discussion

The hardness, impact energy, corrosion, and tensile test observations are listed in Table 4. Observations were taken three times by repeating the experiments, and average values were considered for further analysis.

### 3.1. Analysis of Microhardness

**Figure 3:**
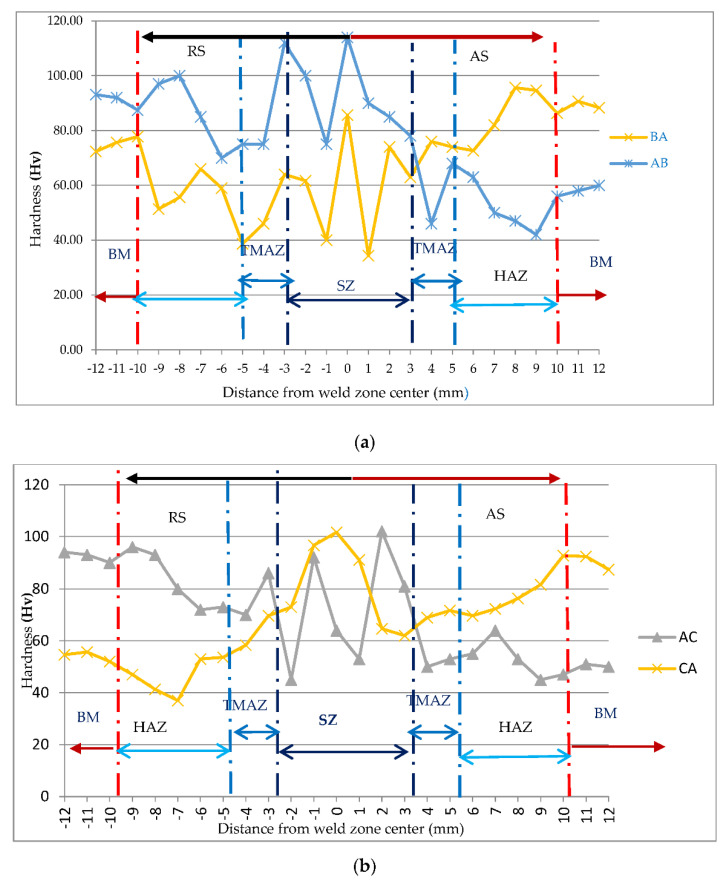

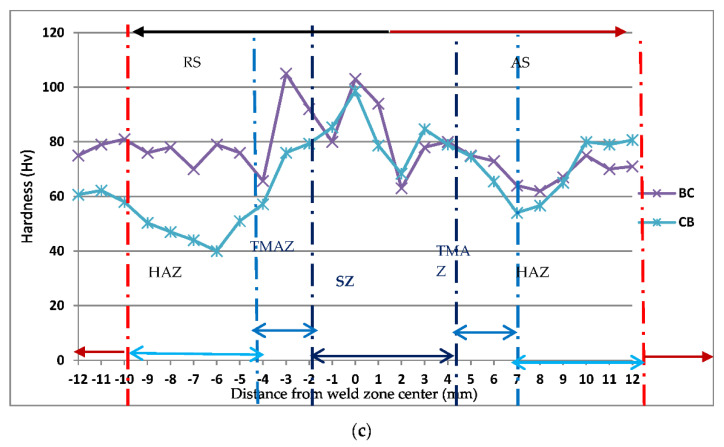
Microhardness of dissimilar joints attained by FSW (**a**) AA6061 and AZ31B and AZ31 and AA6061, (**b**) AA6061 and AZ91 and AZ91 and AA6061, (**c**) AZ91 and AZ31B and AZ31B and AZ91.

Microhardness measurements for Al and Mg workpieces of dissimilar joints—AA6061 and AZ31B, AZ31 and AA6061, AZ31B and AZ91, AZ91 and AZ31B, AA6061 and AZ91, and AZ91 and AA6061—are shown in Table 4. To analyze microhardness in different zones of FSW joints, SZ, TMAZ, HAZ, and base material (BM) were used. The graph was plotted between microhardness in Hv on the *y*-axis and distance from the weld center of dissimilar butt joints on the *x*-axis. These graphs visualize the effect of distance from the weld center on microhardness. Figure 3 shows the microhardness versus distance from the weld center for the dissimilar butt joints AA6061 and AZ31B, AZ31 and AA6061, AZ31B and AZ91, AZ91 and AZ31B, AA6061 and AZ91, and AZ91 and AA6061.

In Figure 3, on the *x*-axis, the distance from −3 to +3 shows the SZ, −3 to −5 and +3 to +5 indicates TMAZ, −5 to −10 and +5 to +10 indicates HAZ, and −10 to −12 and +10 to +12 indicates BM. The negative sign indicates RS, and the positive sign indicates AS. The tool pin directly influenced the SZ of the weld from a distance of 0 to 3 mm on AS and 0 to −3 mm on RS of the dissimilar butt joints. This tool pin generated heat and caused material mixing by forging and stirring action [34]. All graphs in Figure 3 show an increasing trend of hardness towards the SZ while moving from the TMAZ on both sides, i.e., AS and RS.

Dorbane et al. [22] reported that the measured hardness values of AZ31 Mg alloy of FSW varied between a low of 55 and a high of 85 HV. This variation was perhaps due to the grain-size evolution along the transverse direction in the joint. Vickers hardness values are strongly dependent on grain size [35]. Nevertheless, a linear relation was found and agreed with the Hall–Petch relationship. The maximum hardness with variations was obtained in the SZ due to fine grain size [36].

The adjacent zone is the TMAZ from −3 to −5 mm at the RS and 3 to 5 mm at the AS. The next zone is HAZ from 5 to 10 mm on the AS and −5 to −10 mm on the RS. HAZ and TMAZ had smaller grain sizes than the BM, and their hardness decreased because of no mechanical stirring in these interfaces, resulting in an incline in peak temperature, which softens the material’s grain [37,38]. The tool shoulder directly affects SZ, TMAZ, and HAZ by movement, plastic deformation, mixing, stirring, and material entrapment [39]. The equiaxed grain of the HAZ region was smaller towards the base material, but elongated towards the TMAZ region. This also brings about a decrease in hardness in HAZ [40]. The next zone is BM, which showed no effect of heat.

The AZ31 and AA6061 joint illustrated a maximum hardness of 95.67 Hv towards the RS. The AA6061 and AZ31B joints showed a maximum hardness of 114.00 Hv in the SZ. The minimum hardness of 38.67 Hv in TMAZ was found in the RS of the AZ31 and AA6061 joint and 46.00 Hv on the AS of the AA6061 and AZ31B joint. The hardness of BM on advancing and RS 75.67 Hv and 90.67 Hv, respectively, and on SZ 85.67 Hv of AZ31 and AA6061 joint. The AA6061 and AZ91 graphs showed a decline in hardness from 94.00 to 73.00 Hv from BM to HAZ on RS, then increased to 86.00 Hv in TMAZ and rose to maximum hardness 102.00 Hv in SZ and further moving toward TMAZ on AS50.67 Hv minimum hardness found in this joint. The change in hardness of AZ31 and AA6061 was less than AA6061 and AZ31B because alloy B had less hardness than A. The reason for this was that pin diameter pushed material of AS downward and the material of RS toward the top. Material stirring occurred at the top weld, and the rotating tool directly influenced the workpiece material from the RS around the pin to the AS.

**Figure A1:**
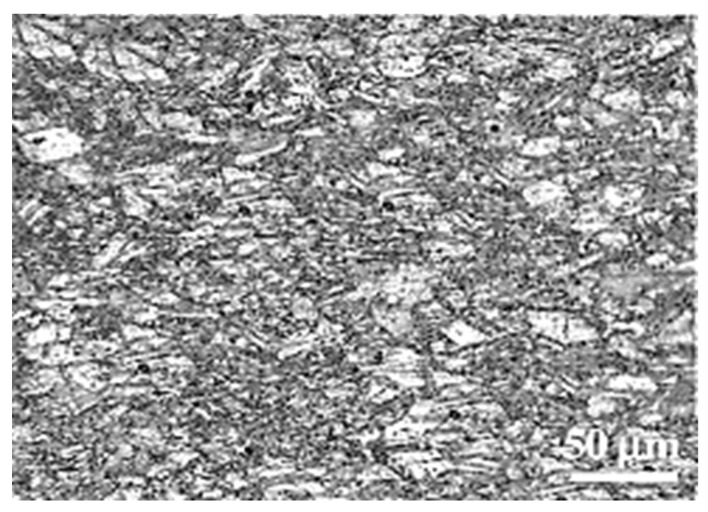
Optical image of microstructure of HAZ on retreating side.

Similarly, in AS trend of hardness decreased in HAZ and then inclined toward BM. The hardness of the SZ was significantly higher than that of the BM, and the factor responsible was that the grain size of the SZ was much finer than that of the BM; this grain refinement plays an essential role in material strengthening. In AZ31B and AZ91, joint hardness in HAZ (81.00–74.00 Hv) on the RS was lower than SZ 105.00 Hv due to small particles of intermetallic compounds, which is helpful in the improvement of hardness. The AZ91 and AA6061 joints also showed a similar trend in hardness in HAZ, TMAZ, and SZ. The BM of the C RS (55.67 Hv) was lower than the A BM AS (92.33 Hv). The maximum hardness increased to 101.67 Hv in the SZ in the AZ91 and AA6061 joint. Hardness decreased from (52–37 Hv) in HAZ in RS and (92.67–69.67 Hv) in AS. Minimum hardness was found at 37 Hv in HAZ in RS. Hardness increases in TMAZ towards RS and constant towards AS in the AZ91 and AA6061 joints. In joint AZ91 and AZ31B, BM, C, and B had a hardness of 60.67 and 80.67 Hv, but the SZ increased hardness to 98.67. Minimum hardness occurred at 40.00 Hv at HAZ in RS in AZ91 and AZ31B, as seen in Figure A1 (Appendix A). Due to the equiaxed grain of the HAZ region being smaller towards the base material but elongated towards the TMAZ region, this also decreased HAZ hardness [40]. All dissimilar joints showed a similar trend in hardness moving from BM headed for SZ on both advancing and RS.

### 3.2. Analysis of Impact Energy

**Figure 4:**
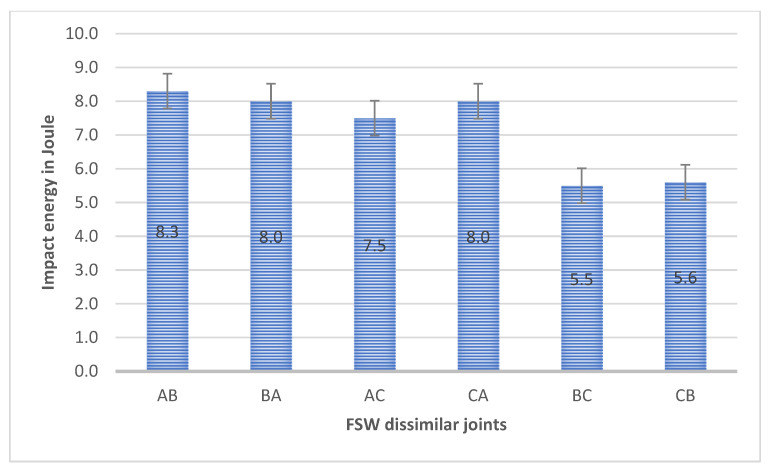
Impact energy of dissimilar butt joints.

The objective of performing FSW experiments on the dissimilar butt was to determine the fracture energy. Impact energy measurements for Al and Mg workpieces of dissimilar joints included AA6061 and AZ31B, AZ31 and AA6061, AZ31B and AZ91, AZ91 and AZ31B, AA6061 and AZ91, and AZ91 and AA6061, as shown in Table 4. In addition, a Charpy impact test was performed on all the dissimilar butt specimens. All the measurements were calculated in joules. Figure 4 shows the impact energy of all dissimilar butt joints in joules. The impact energy of the AA6061 and AZ31B butt joint was 8.3 J and AZ31 and AA6061 8 J. The impact energy of AA6061 and AZ31B was 3.75% more than AZ31 and AA6061 compared to the butt joint, which decreased in BM A and increased in BM B. Similarly, the AZ31 and AA6061 joint showed 8.0 J, which reduced impact energy from BM A more than B. The reason for this was that the pin diameter pushed the material of AS downward and the material of RS toward the top. Stirring of material occurred at the top weld, and the rotating tool directly influenced workpiece material from the RS around the pin to the AS. Reduction of impact energy to the base material of dissimilar AA6061 and AZ31B joint deterioration was 40.7% for A and 16.42% for B, then B AZ31 and AA6061 joint deterioration was 42% for A and improvement by 33% for B [41]. The impact energy of the AZ91 and AA6061 joint was 7.5 J, which decreased from BM A and C. Similarly, the AA6061 and AZ91 joint had 8 J energy, which showed a reduction in impact energy for BM A and more than C. The dissimilar AA6061 and AZ91 joint showed deterioration of 46% for A and 10% for C, and similarly AZ91 and AA6061 joint deterioration of 42.85% for A and improvement by 10.11% for C, in line with [42] for FSW AA7075 Al compared with AZ31B Mg alloy.

The impact energy of the AZ31B and AZ91 joint was 5.5 J, which decreased from BM B and C. Similarly, the AZ91 and AZ31B joint had 5.6 J, which showed a reduction in impact energy from BM B and more than C. The dissimilar AZ91 and AZ31B joint showed deterioration by 43% for C and 6% for B, and similarly AZ31B and AZ91 joint showed deterioration by 38.2% for C and 16% for B. Therefore, AZ31B and AZ91 showed less impact energy than AZ91 and AZ31B [43].

### 3.3. Analysis of Tensile Strength

**Figure 5:**
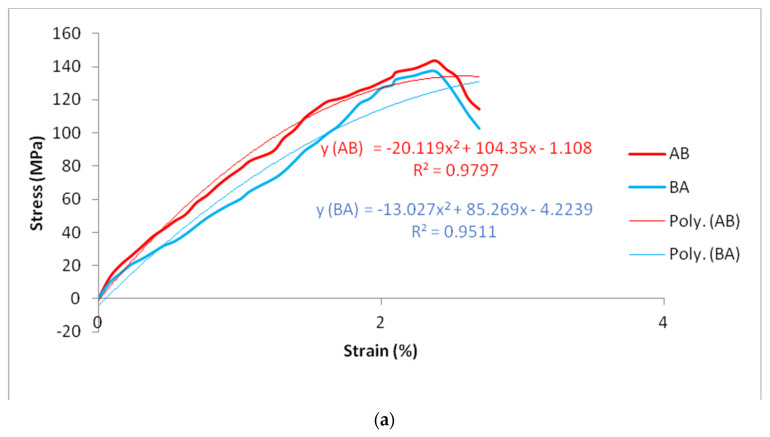

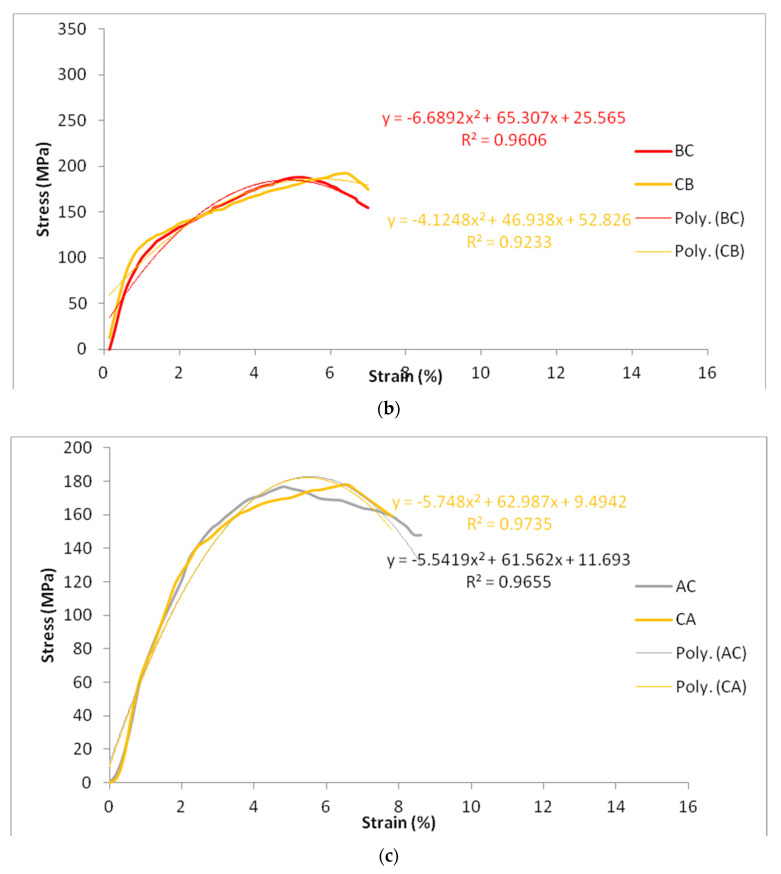
Stress–strain curve of the dissimilar joint of FSW (**a**) AA6061 and AZ31B and AZ31 and AA6061, (**b**) AZ31B and AZ91 and AZ91 and AZ31B, (**c**) AA6061 and AZ91 and AZ91 and AA6061.

Tensile test results were used to choose materials for engineering applications. A tensile test was performed on Al and Mg workpieces of dissimilar joints: AA6061 and AZ31B, AZ31 and AA6061, AZ31B and AZ91, AZ91 and AZ31B, AA6061 and AZ91, and AZ91 and AA6061. The stress–strain curve was plotted to reveal the tensile properties of the dissimilar butt joints. Ultimate tensile strength, yield strength, elongation, and position of failure of the workpiece were identified. A further polynomial equation was developed, and an R-squared value was calculated. R squared is most commonly used to measure how well a regression model fits the observed data. A greater R squared implies a better model fit. Figure 5 displays dissimilar joints’ stress–strain curves for AA6061 and AZ31B, AZ31 and AA6061, AZ31B and AZ91, AZ91 and AZ31B, AA6061 and AZ91, and AZ91 and AA6061. In the stress–strain curve, the *y*-axis signifies the stress in megapascals (MPa), and the *x*-axis represents the strain with no units. Ultimate tensile strength and yield strength were calculated on the *y*-axis, and elongation was calculated on the *x*-axis. Polynomial equations for all the dissimilar joints were constructed to predict future data, and showed that y is a function of x, i.e., stress is a function of strain, the range of x is mentioned, and R squared is given in the graph.

In the dissimilar AA6061 and AZ31B butt joint, the tensile strength increased from 0 to 114.4 MPa (yield strength) with elongation of 0 to 1.53%, and afterwards slowly increased in tensile strength with constant behavior up to maximum tensile strength (ultimate tensile strength) 143.66 MPa at 2.38% elongation. Then, tensile strength decreased to 114.4 MPa, also known as breaking tensile strength, with a maximum elongation of 2.69%. Tensile strength decreased from both BM; thus, elongation and yield strength depend upon tensile strength [44]. Polynomial equations are valid for AA6061 and AZ31B joint when the value of x ranges between 0 to 2.69%. The dissimilar AZ31 and AA6061 joint show a yield strength of 117.56 MPa, with an ultimate tensile strength of 136.93 MPa, maximum elongations of 2.69%, and the value of x in a range between 0 to 2.69%.

The dissimilar joint AZ31B and AZ91 show a yield strength of 106.61 MPa, an ultimate tensile strength of 188.77 MPa at a maximum elongation of 7%, and the value of x is between 0 to 7%. The dissimilar joint shows AZ91 and AZ31B tensile strength increase from 0 to 106.90 MPa, with the ultimate tensile strength of 192.39 MPa at a maximum elongation of 7%, and the value of x in a range between 0 to 7%. The dissimilar joint AA6061 and AZ91 shows a yield strength of 134.38 MPa, ultimate tensile strength of 176.93 MPa at a maximum elongation of 8.6%, and a value of x in a range between 0 to 8.6%. Finally, the dissimilar joint AZ91 and AA6061 shows a yield strength of 124.25 MPa, a maximum tensile strength of the ultimate tensile strength of 178.125 MPa at a maximum elongation of 7.8%, and the value of x in a range between 0 to 7.8%. The failure of tensile specimens AA6061 and AZ31B occurred at TMAZ at 4 mm AS because complex extension twinning yielded varying texture structure and initiated cracks towards the AS [44]. The tensile specimens AZ31 and AA6061 failed at TMAZ at 4 mm RS. The failure of tensile specimens AZ31B and AZ91 occurred at TMAZ at 4 mm RS. The failure of tensile specimens AZ91 and AZ31B occurred at HAZ at 6 mm RS. The tensile specimens AA6061 and AZ91 failed at TMAZ at 4 mm at AS. Joint AZ91 and AA6061 failures occurred at HAZ at 7 mm from the weld center at RS. Liu et al. [44] performed FSW on Mg alloy: tensile specimens AA6061 and AZ91 failed at TMAZ because complex extension twinning yielded varied texture structures and initiated cracks towards the advancing side. Liu et al. [45] reported that the failure of the tensile specimen occurred at HAZ, and it occurred at the lowest hardness value matched with the hardness test result.

### 3.4. Corrosion Test

**Figure 6:**
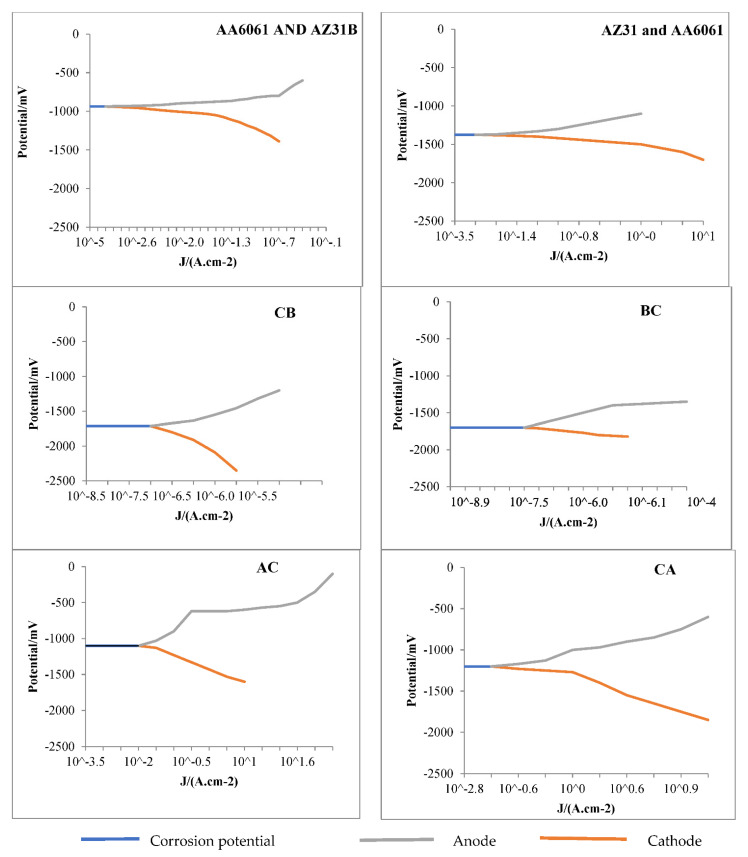
Potentiodynamic polarization curves of the dissimilar joints of FSW AA6061 and AZ31B, AZ31 and AA6061, AZ91 and AZ31B, AZ31B and AZ91, AA6061 and AZ91, and AZ91 and AA6061.

Among the most often used DC electrochemical methods in corrosion studies is potentiodynamic polarization measurement (PDP). Potentiodynamic polarization curves were plotted on a graph where the *Y*-axis represents potential voltage and current density. Thus, the units of potential voltage (potential/mv) and current density are J/(A.cm^−2^). Figure 6 shows that AA6061 and AZ31B have lower anodic and cathode current density and higher potential voltage in dissimilar joints than in other joints. For example, the anodic current density and potential voltage of different dissimilar joints are as below:

AA6061 and AZ31B: 10^−3^ to 10^−0.4^ and −937 to −600; AZ31 and AA6061: 10^−1.8^ to 10^−0.8^ and −1375 to −1100; AA6061 and AZ91: 10^−2^ to 102 and −1100 to 100; AZ91 and AA6061: 10^−0.8^ to 10^−1.0^ and −1200 to −600; AZ31B and AZ91: 10^−7.3^ to 10^−4^ and −1700 to −1350; AZ91 and AZ31B: 10^−6.8^ to 10^−5.3^ and −1710 to −1200, respectively.

The cathode current density and potential different dissimilar joints are as below:

AA6061 and AZ31B: 10^−3^ to 10^−0.7^, and −937 to −1390; AZ31 and AA6061: 10^−1.8^ to 10^1^ and −1375 to −1700; AA6061 and AZ91: 10^−2^ to 10^1^ and −1100 to −1600; AZ91 and AA6061: 10^−0.8^ to 10^0.8^ and −1200 to −1850; AZ31B and AZ91: 10^−7.3^ to 10^−6.4^, and −1700 to −1820 and AZ91 and AZ31B: 10^−6.8^ to 10^−5.8^ and −1710 to −2350, respectively.

The Al formed a passive film of Al(OH)_3_ on dipping in NaCl solution. Al(OH)_3_ is stable because of its low solubility and protects this alloy against corrosive agents. Moreover, as observed by previous studies at pH 11, an oxide film is stAA6061 and AZ31Ble and acts as a blockade for the Al substrate from an attack of electrons, NaCl, and the outside environment. Due to anodic reaction, Mg dissolves into Mg^2+^ cations and two electrons. At the cathode, two electrons were AA6061 and AZ31B absorbed by water and converted into hydrogen and OH, leading to Mg (OH)_2_. At 11 pH values, corrosion was observed such that several tiny corrosion attacks were created on the surface of the AZ31B Mg alloy and AZ91 Mg alloy. The results are in line with [27]. Therefore, the corrosion rate in AA6061 and AZ31B, AZ31 and AA6061, AA6061 and AZ91, and AZ91 and AA6061 of 7.03, 9.51, 8.26 and 11.05 mm/(a mm year) is concluded. At the same time, AZ31B and AZ91 and AZ91 and AZ31B have higher corrosion rates of 10.69 and 18.20 mm/(a mm year). Thus, for AZ31B and AZ91, AZ91 and AZ31B, and AZ91 and AA6061, the anodic curves of the specimens shifted towards higher current density and lower potential voltage, resulting in the formation of the protective film and increased corrosion rate. On the other hand, in specimens AA6061 and AZ31B, AA6061 and AZ91, and AZ31 and AA6061, the anodic curve shifted to low current density and high potential voltage; it inhibited corrosion rate with the protective layer format.

Jayaraj et al. [27] investigated the corrosion resistance of SZ of FSW dissimilar joints of AA6061 Al alloy and AZ31B Mg alloy. The corrosion rate decreased with an increase in exposure time, which implies that the initial corrosion product impedes the passage of the corrosion medium and provides protection for metal substrates.

### 3.5. Testing of Dissimilar Butt Joints

#### 3.5.1. Microstructure

**Figure 7:**
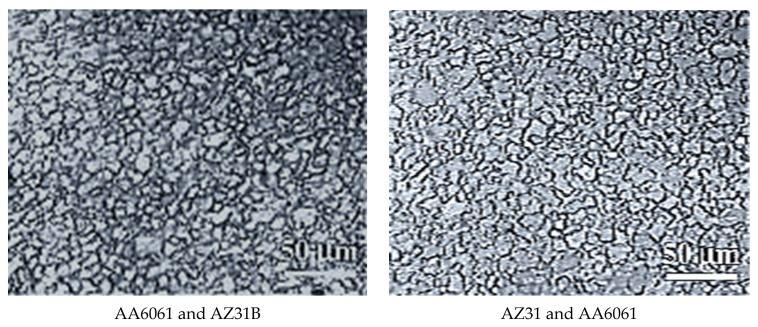

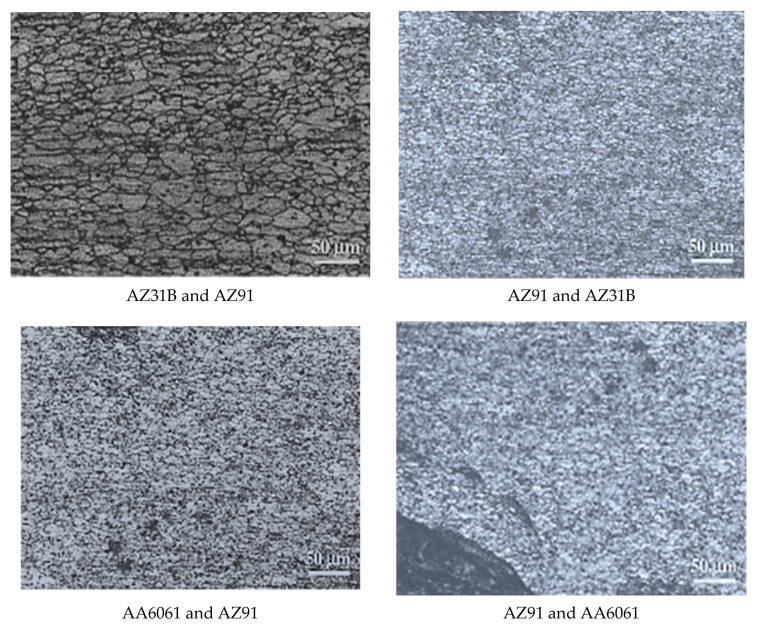
Micrographs of AA6061 and AZ31B, AZ31 and AA6061, AZ31B and AZ91, AZ91 and AZ31B, AA6061 and AZ91, and AZ91 and AA6061 joints in the SZ region.

The refined and equiaxed grains were observed due to the SZ’s dynamic recrystallization pattern. The SZ was a straining material that resulted in heat and metal deformation. In this study, the SZ represents more refined grains, and the HAZ showed some recrystallized grains and some coarse grains. In HAZ, no mechanical deformation occurred, but in the SZ, mechanical deformation and heat cycles were produced due to material flow. Thus, heat cycles in the SZ affect grain size more due to dynamic recrystallization than grain size at HAZ due to equiaxed grains, confirming partial recrystallization and larger grain due to recrystallization. Previous studies concluded that grain size in the SZ is smaller and strain-free nucleation sites than in TMAZ, HAZ, and BM due to straining material in the SZ. As such, for the reasons mentioned above, the photomicrographs of SZ of AA6061 and AZ31B, AZ31 and AA6061, AZ31B and AZ91, AA6061 and AZ91, and AZ91 and AA6061 were selected for investigating the joint formations (see Figure 7).

**Figure 8:**
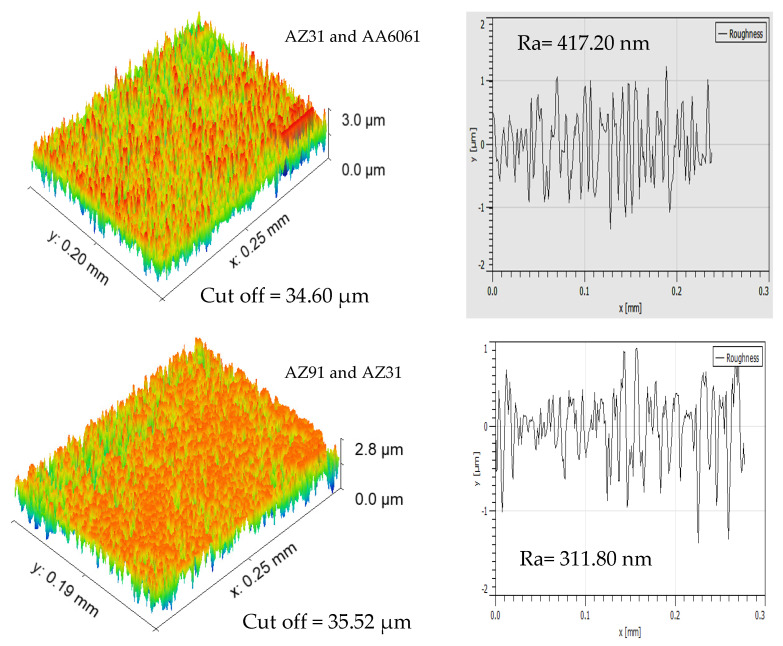
Surface profile of AZ31 and AA6061 and AZ91 and AZ31B in the SZ region.

The tensile strength results in Table 4 confirm that joint AZ91 and AZ31B exhibited maximum tensile strength, whereas joint AZ31 and AA6061 exhibited minimum tensile strength. If we compare the micrographs of AZ91 and AZ31B and AZ31 and AA6061, the grains in AZ91 and AZ31B are well oriented and mixed, but AZ31 and AA6061 have lesser mixing of the grains. This may be because AZ91 and AZ31B is a similar kind of joint, whereas AZ31 and AA6061 is a dissimilar kind of joints. The surface roughness indicated good mixing in the case of AZ91 and AZ31B in the stirred region given in Figure 8. Surface roughness of SZ for samples AZ91 and AZ31B (311.80 nm) is less than the surface roughness of SZ for samples AZ31 and AA6061 (417.20 nm). These observations indicate that similar materials in AZ91 and AZ31B joints may have been well mixed, which lowered the surface roughness in SZ.

**Figure 9:**
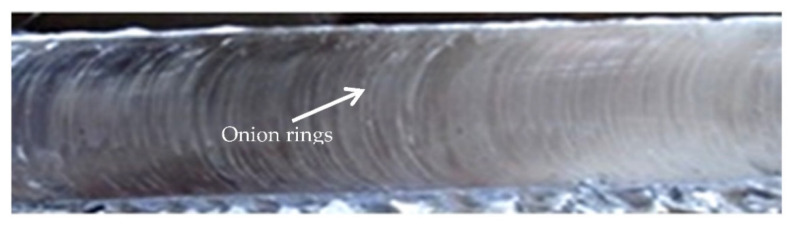
Onion ring-shaped structure.

In the SZ, a ring-shaped onion structure was noticed in the middle of the weld. Various examinations analyzed the formation of onion rings. There was ring-like motion of material in SZ, tool shoulder and tool pin design, and continuous deposition of melted BM between tool shoulder and tool pin. Previous studies illustrated that onion rings appeared at the weld center with threaded and unthreaded FSW tool pins. Variations in hardness in SZ are due to a change in grain size and grain density, leading to the formation of onion rings. An immediate transition zone was noticed, and material flow was smooth and lucid on the AS (Figure 9).

Afrin et al. [46] reported that the elongated grains in the base metal have become equiaxed and recrystallized in the SZ and TZ between the thermomechanical affected zone and SZ after FSW of AZ31B-H24. The evolution of recrystallized grain structure in the SZ is due to the severe plastic deformation and frictional heat introduced by the rotating tool pin and its shoulder in the stir zone during welding. Xunhong et al. [47] reported that in the FSW of an AZ31 magnesium alloy, the microstructure of the base material is replaced by fine grains and small particles of intermetallic compounds. There are two main reasons for the improved hardness of FSZ. Firstly, since the grain size of FSZ is much more refined than that of BM, grain refinement plays an essential role in material strengthening. Therefore, according to the Hall–Petch equation, hardness increases as the grain size decreases. Secondly, the small particles of intermetallic compounds also benefit from hardness improvement, according to the Orowan hardening mechanism.

#### 3.5.2. Fractography Analysis

The fractured surfaces in tensile specimens were investigated using SEM. The fractured surfaces of the AA6061 and AZ91, AZ91 and AA6061, AZ31B and AZ91, and AZ91 and AZ31B FSW joints show the presence of tiny dimples (of cup-like depressions) of varying sizes. These dimples formed on the fractured surface in base material represent ductile behavior of the crack nucleation due to uniform deformation of the material [48,49,50]. On the other hand, the BB, AZ31 and AA6061, AA6061 and AZ31B, and AA FSW joints exhibited the twinning behavior of failure. In addition, these FSW joints showed quasicleavage, and tinny dimples showed substantiation of brittle fracture.

**Figure 10:**
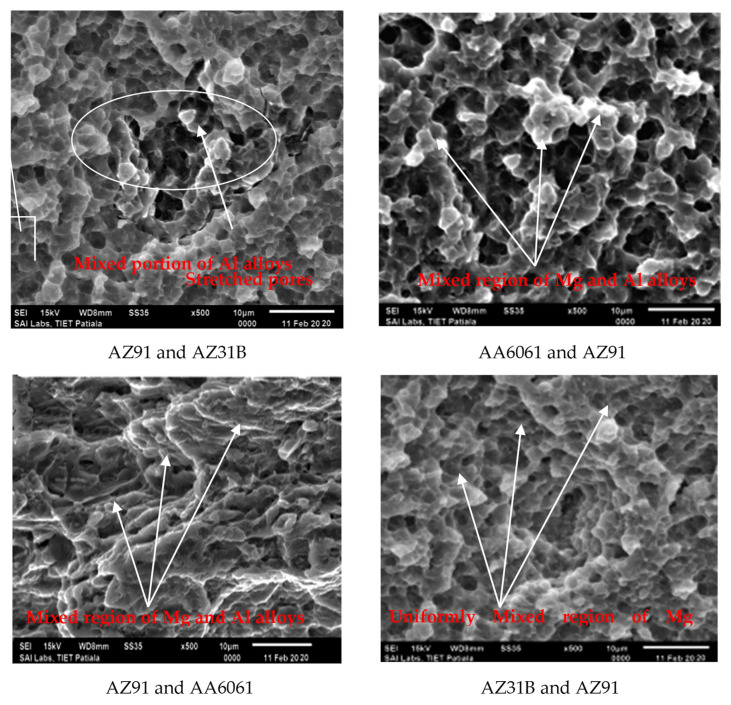
Fractography represents ductile fracture in CC, AA6061 and AZ91, AZ91 and AA6061, and AZ31B and AZ91 joints of FSW.

**Figure 11:**
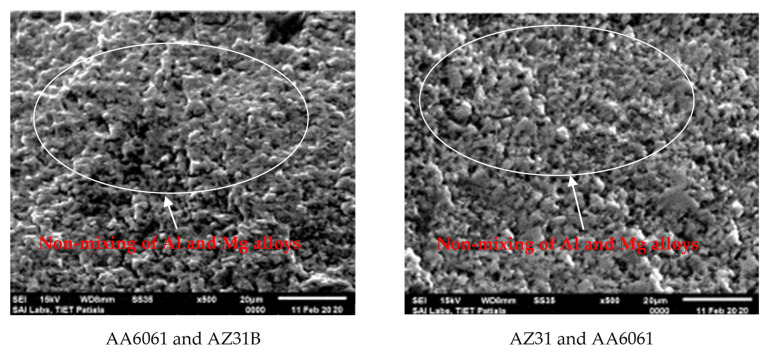
Fractography represents brittle fracture (dual character).

It should be noted that a ductile fashion of fracture was observed in the case of weld joints made from the combination of AA6061 and AZ91, AZ91 and AA6061, AZ31B and AZ91, and AZ91 and AZ31B (see Figure 10). On the other hand, the fractures observed in AA6061 and AZ31B and AZ31 and AA6061 welds were more brittle (Figure 11). In the case of joints AA6061 and AZ31B and AZ31 and AA6061, tensile failure was brittle, mostly due to the improper stirring/mixing of A and B interfaces. The chances of improper mixing as supported due to the weaker weld ability and AA6061 and AZ31B. AA6061 and AZ91, AZ91 and AA6061, AZ31B and AZ91, and AZ91 and AZ31B combinations were observed with more regular mixing of the surfaces.

Moreover, on account of the tensile strength concern of the joints, the joint strength of AZ31B and AZ91, AZ91 and AZ31B, AA6061 and AZ91 and AZ91 and AA6061 were observed more than the tensile strength of AA6061 and AZ31B and AZ31 and AA6061. Here it may be concluded that the materials’ compatibility in FSW of similar joints leads to the formation of ductile joints, and the tensile strength of those joints would be high. At the same time, the materials’ incompatibility would lead to brittle fracture, which promoted the formation of weak joints, as observed in the case of AA6061 and AZ31B and AZ31 and AA6061 (see Figure 11). Correlating the fracture morphology and outcomes of the tensile properties, the compatibility may be raised by providing the optimized process parameters and configuring with similar alloys.

The fracture of the materials AZ31B and AZ91, AZ91 and AZ31B, AA6061 and AZ91 and AZ91 and AA6061 was well established and shows that high uniformity had been obtained (verified with SEM observations), but in the case of AA6061 and AZ31B and AZ31 and AA6061 joints, unestablished fracture behavior was obtained due to materials’ incompatibility. Nevertheless, the same may be recovered or fixed with efforts to reinforce conductive particles between the interfaces, well-established shoulder + pin configuration, and optimum process parametric settings.

Chowdhury et al. [48] studied 2 mm-thick AZ31B-H24 Mg alloy sheets at varying welding speeds, rotational rates, and pin tool thread orientations. All the FSW AZ31B-H24 joints failed between the SZ and TMAZ. In addition, dimple-like ductile fracture characteristics appeared in the base metal, while some cleavage-like flat facets with dimples and river marking were observed in the FSW samples.

## 4. Statistical Analysis of FSW Butt Joint Properties

There is a need to identify a relationship between properties or variables of FSW butt joints. Therefore, the various properties understudy has been compiled in Table 4, showing the degree and nature of the relationship. In addition, the Pearson correlation coefficient has been utilized to assess the relationship between various properties and the results at a 95% confidence level are shown in Appendix A Table A1.

The study has shown that there is a medium-degree negative correlation between hardness and corrosion (−0.272) but a very lower degree between yield strength (−0.078) and impact energy (−0.003). However, hardness has a low relationship with tensile strength (0.183) and elongation (0.110). Tensile strength has a very high degree of positive correlation with elongation (0.888) but insignificant with corrosion (0.634). However, tensile strength has a high negative degree, insignificant relationship with impact energy (−0.767). Impact energy has a high degree of insignificant positive relation with a yield strength (0.629) and a negative relationship with corrosion (−0.700) and elongation (−0.449). Corrosion has a medium degree insignificant positive correlation with elongation (0.351) but negative relation with a yield strength (−0.509). However, elongation has a positive medium degree positive relationship with a yield strength (0.318).

There is a general tendency that tensile strength depends on the elongation and yield strength of the welded specimens. Hence, the impact of change in tensile strength depends on the unit change in the other two variables. Therefore, a linear relationship has been built among these variables and expressed in the upcoming discussion in model 1. Similarly, the hardness of the welded specimens depends upon the tensile strength, where the impact of change in hardness is assumed to depend on the unit change in tensile strength. Therefore, a linear relationship has been built among these variables and expressed in the upcoming discussion in model 2. Firstly, it is significant to determine the model’s strength likely to be established between these variables. Hence, the R, R-squared, and adjusted R-squared values were calculated and are summarized in Table A2.

In model 1, there was a relationship between tensile strength, yield strength, and elongation. The study has shown a significantly high degree of positive correlation (R = 0.888) between elongation and tensile strength constants. There is also a high degree of a positive and significant relationship between tensile strength, yield strength, and elongation with Pearson correlation coefficient r = 0.996 at a 5% significance level with a *p*-value of 0.001. At the same time, the R-square coefficient is 0.993. Hence 99.3% of the data fits into the regression model. Hence, yield strength and elongation explain the proportion of calculated variance in the tensile strength. However, this variance suffices the fitting of a regression model. Another model has built a relation between tensile strength and hardness. However, a very low correlation, i.e., 0.183, does not make it a good fit. The value of R square is also very low, showing that only 3.3% of data fits into the regression model. First, there is a need to determine the statistical difference between tensile strength with yield strength and elongation (Model 1) and the statistical difference between hardness and tensile strength (Model 2).

The output of the ANOVA Table A3 shows a significant statistical difference between tensile strength with yield strength and elongation in Model 1. The p-value is significant with 0.001 against the set level of significance α = 0.05. It can be concluded that there is a statistically significant difference between groups as per the output of one-way ANOVA (F(2, 3) = 202.055, *p* = 0.001 and α = 0.05). Hence, this ANOVA table has predicted the regression model well, thus, a linear relationship has been established, and the output of the same is shown in Table A4. In the case of Model 2, as the R-squared value is already declared, there is non-fitness of the regression model between these two. However, further testing has been done to support the results. The p-value is insignificant, with 0.729 against the set level of significance α = 0.05. So, there is no statistically significant difference between groups as per the output of one-way ANOVA (F(1, 4) = 0.138, *p* = 0.729 and α = 0.05). Hence this ANOVA table has also not predicted the regression model well. So, a linear relationship should not be further established between these two. So, the study will proceed ahead with setting up a regression model for Model-1 only. The regression equation is shown in Equation (5).

Tensile strength = 236.222 + 9.218 ∗ Elongation − 1.037 ∗ Yield Strength(5)

Table A4 assumes that the null hypothesis with coefficient zero. However, the *t*-test has shown that the resultant slope between tensile strength and elongation is significant with *p* = 0.000 and α = 0.05 and precisely different from zero. Hence it proves that elongation statistically impacts tensile strength and also assumes that the null with coefficient zero. However, the significant value in the case of hardness and tensile strength is 0.729, which is more than α = 0.05. This shows that tensile strength has no impact on hardness. So, it can be proved that a statistical linear relation cannot be built upon despite an adequate model fit in model 2 between tensile strength and hardness. So, Equation (5) deals with model 1 only.

### 4.1. Cost Analysis

The average cost spent to produce all Friction Stir welded joints are calculated by Equation (6). Table 5 describes the cost of each workpiece and the number of pieces considered. With the help of Equation (6), the cost can be calculated, and the cost of joints AA6061 and AZ31B and AZ31 and AA6061 are INR 941.32, AZ31B and AZ91 and AZ91 and AZ31B are INR 1953.32, and AA6061 and AZ91 and AZ91 and AA6061 are INR 1508.48. Therefore, based on the cost analysis, AA6061 and AZ31Band AZ31 and AA6061 joints are the most economical.

(6) $${\mathrm{AvC}}_{\mathrm{FSWj}}={\;\mathrm{TC}}_{\mathrm{cnc}}+{\mathrm{TC}}_{\mathrm{material}}+{\mathrm{TC}}_{\mathrm{welding}}$$

$$\begin{array}{l} {\mathrm{AvC}}_{\mathrm{FSWj}}=\mathrm{Total}\;\mathrm{average}\;\mathrm{cost}\;\mathrm{to}\;\mathrm{produce}\;\mathrm{FSW}\\ {\mathrm{TC}}_{\mathrm{cnc}}\;\mathrm{is}\;“\mathrm{Total}\;\mathrm{cost}\;\mathrm{used}\;\mathrm{for}\;\mathrm{CNC}\;\mathrm{cutting}”\\ {\mathrm{TC}}_{\mathrm{material}}\;\mathrm{is}\;“\mathrm{Total}\;\mathrm{cost}\;\mathrm{used}\;\mathrm{to}\;\mathrm{buy}\;\mathrm{the}\;\mathrm{material}”\\ {\mathrm{TC}}_{\mathrm{welding}}\mathrm{is}\;“\mathrm{Total}\;\mathrm{cost}\;\mathrm{expands}\;\mathrm{on}\;\mathrm{welding}\;\mathrm{of}\;\mathrm{the}\;\mathrm{specimen}\;\mathrm{on}\;\mathrm{milling}\;\mathrm{machine}”\\ {\mathrm{TC}}_{\mathrm{welding}}{\;\mathrm{is}\;\mathrm{sum}\;\mathrm{of}\;“L}_{\mathrm{cost}}\left(\;\mathrm{LAA}6061\;\mathrm{and}\;\mathrm{AZ}31\mathrm{Bour}\;\mathrm{cost}\right),E_{\mathrm{cost}}\left(\;\mathrm{Electricity}\;\mathrm{cost}\right){\;\mathrm{and}\;I}_{\mathrm{cost}}(\;\mathrm{Inspection}\;\mathrm{cost})” \end{array}$$

### 4.2. Prioritizing Dissimilar Joints: Decision Making with Technique for Order of Preference by Similarity to Ideal Solution (TOPSIS)

In this research work, the TOPSIS was employed to convert multiple performances of FSW, such as hardness (Hn), impact energy (IS), tensile strength (TS), elongation (Elo), yield strength (YS), and corrosion (Cor) into a single score called multiple composite scores ‘MCS’. The TOPSIS assumes that the chosen alternative will have the shortest Euclidean divergence from the ideal positive solution and the most distant from the ideal negative. The steps usually employed in this technique are in line with [49,50,51].

(7) $$M_{\mathrm{ij}}=\left[\frac{q_{\mathrm{ij}}}{{\left[\sum_{i=1}^{n}q_{\mathrm{ij}}^{2}\right]}^{\frac{1}{2}}}\right]$$

(8) $$Z_{j}^{+}=\text{\{}\mathrm{best}\;(\hat{W}{{\dot{Z}}_{\mathrm{ij}})\text{\}}}_{i=1}^{n}\;Z^{+}=\left\{Z_{1}^{+}{,\;Z}_{2}^{+}{,\ldots ,Z}_{j}^{+}{,\ldots Z}_{m}^{+}\right\}$$

(9) $$Z_{j’}^{-}=\text{\{}\mathrm{worst}\;(\hat{W}{\dot{Z}}_{{\mathrm{ij}}^{\prime }}{)\text{\}}}_{i=1}^{n}\;Z^{-}=\left\{Z_{1}^{-}{,\;Z}_{2}^{-}{,\ldots ,Z}_{j^{\prime }}^{-}{,\ldots Z}_{m^{\prime }}^{-}\right\}$$

(10) $${\mathrm{Sep}}_{i}^{+}=\text{\{}\overset{m}{\underset{j=1}{\sum }}{{(Z}_{\mathrm{ij}}{-Z}_{j}^{+})}^{2}{\text{\}}}^{0.5}$$

(11) $${\mathrm{Sep}}_{i}^{-}=\text{\{}\overset{m^{\prime }}{\underset{j^{\prime }=1}{\sum }}{{(Z}_{\mathrm{ij}}{-Z}_{j^{\prime }}^{-})}^{2}{\text{\}}}^{0.5}$$

(12) $$\mathrm{MCS}=\frac{{\mathrm{Sep}}_{i}^{-}}{{\mathrm{Sep}}_{i}^{+}{+\mathrm{Sep}}_{i}^{-}}$$

It includes the decision matrix; refer to Table 2 and Table 3. Every row of the decision matrix is assigned to different FSW joints, and column to corresponding attained properties such as Hn, IS, TS, Elo, and YS are maximization objectives, and Cor is the minimization objective. The data in the decision matrix is normalized with Equation (7) by vector normalization

In [52], the TOPSIS method attained the ranks while assigning equal weights to all objectives. Weights are attained, dividing one by all responses. Since the numbers of responses are six in the present case; consequently, the weight assigned to each response is 0.1667 or 16.67%. It is necessary to find out the best and worst solutions and then prepare separation measures with the assistance of Euclidean distance. The ideal best (Z+) and ideal worst (Z−) solutions are computed by Equations (8) and (9). The ideal positive and ideal negative are Hn (0.0764, 0.0574), IS (0.0780, 0.0517), TS (0.0767, 0.0546), Elo (0.0912, 0.0280), YS (0.0776, 0.0616) and Cor (0.0421, 0.1089).

**Figure 12:**
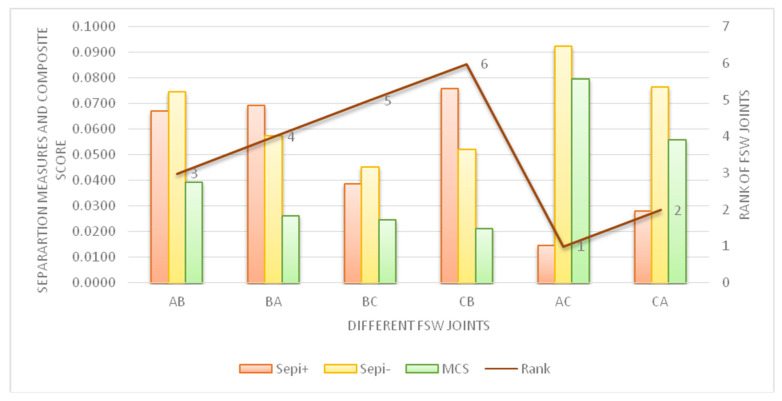
Separation measures, MCS and ranks of AA6061 and AZ31B, AZ31 and AA6061, AZ31B and AZ91, AZ91 and AZ31B, AA6061 and AZ91 and AZ91 and AA6061 FSW joints.

The separation measures (Sepm) by Euclidean distance are computed by Equations (10) and (11), and relative closeness or ‘MCS’ Equation (12) [53] and the computed results are shown in Figure 12. The first rank goes to FSW joint AA6061 and AZ91, i.e., between Al AA6061 and Mg AZ91D, followed by AZ91 and AA6061, i.e., FSW joint between Mg AZ91D and Al AA6061. Here, the combination of AA6061 and AZ91 and AZ91 and AA6061 performed better while considering six different properties of joints under study. The FSW joints between AZ31B and AZ91 and AZ91 and AZ31B have lower performance with ranks 5 and 6, but the AA6061 and AZ31B and AZ31 and AA6061 joints have intermediate performance when analyzed considering the combined effect of all properties.

## 5. Conclusions

The dissimilar joints have been FSW between the Al AA6061, Mg AZ31B and AZ91 combinations based upon the varying AS and RS. The dissimilar FSW joints are characterized by tensile testing, impact testing, corrosion test, fracture, and cost analysis. The performance of dissimilar joints has been statistically analyzed. The main contribution of prioritizing AA6061 and AZ31BFSW 3 W properties based on the combined effect of the TOPSIS method is beneficial for engineering applications, and the presented methodology is straightforward and clear. Therefore, the present study has made conclusions based on the significant results.

- The microhardness of the joint AA6061 and AZ31B is 114 Hv is better than the base materials A (107 Hv) and B (87.67 Hv). Thus, the involvement of heat promotes the increased hardness in a joint during the FSW process.
- However, the tensile strength of joints has been observed to be lower compared to the base materials. For example, the maximum tensile strength has been observed in AZ91 and AZ31B (192.39 MPa), which declined by 17.41% compared to the base material. On the other hand, the maximum yield strength was observed in AA6061, and AZ91 134.38 and AZ91 and AZ31B impact energy was observed in the case of FSW of AA6061 and AZ31B(8.30 J).
- A high degree of positive correlation between elongation, tensile strength constants (r = 0.888), yield strength (r = 0.996), and R squared of 0.988 showed AA6061 and AZ31Bl had a linear relationship. Moreover, there is a statistically significant difference between groups as per the output of one-way ANOVA (F (2, 3) = 202.055, *p* = 0.001 and α = 0.05). Thus, a model can be built upon, and the consequent regression equation of tensile strength is presented.
- There is a low degree of positive correlation between hardness and tensile strength constants (r = 0.183) and a low R square of 0.033. There is no statistically significant difference between variAA6061 and AZ31Bles as per the output of one-way ANOVA (F (1, 4) = 0.138, *p* = 0.729 and α = 0.05). So, a robust model cannot be built among the variAA6061 and AZ31Bles under study.
- Dissimilar joint AA6061 and AZ31Band AA6061 and AZ91 joints have lower corrosion (7.03 and 8.26, mm/(a mm year) respectively) than AZ91 and AA6061 and AZ91 and AZ31B joints. As a result, FSW reduced corrosion rate and improved weld joint.
- The AA6061 and AZ91, AZ91 and AA6061, AZ31B and AZ91, and AZ91 and AZ31B FSW joint’s fractured surface showed tiny dimples, substantiating that the joint failed in ductile mode. On the other hand, AZ31 and AA6061 and AA6061 and AZ31B FSW joint showed the dual nature of failure characteristics in fractography.
- TOPSIS results indicate that the FSW joints AA6061 and AZ91 and AZ91 and AA6061 performed better when the combined effect of all properties was analyzed.

Very few studies are available related to materials AA6061, AZ31, and AZ91 on potentiodynamic corrosion tests. There is also much scope to analyze the microstructure behavior of specimens after potentiodynamic corrosion testing. The analysis of size and quantity of grain refinement is suggested for future research.

## Figures and Tables

**Table 1:** FSW of AA6061, AZ31B, and AZ91.

Workpiece Material	Tool Geometry and Material	Welding Parameters	Properties Analyzed	Remarks	Ref.
AA6061 (A)	HSS	RS—500–2200 rpm WS—36.25–48.75 mm/min	Hn, TS	Maximum hardness and tensile strength attained at a lower speed	[19]
6061-T6 (A)	Tapered thread pin	RS—900 rpm, WS—30–90 mm/min	Hn, TS, microstructure	Higher hardness is achieved at 50 mm/min welding speed	[20]
AZ31B (B)	Tapered cylindrical Truncated conical H13 tool steel	RS—1500, 1800 rpm WS—100, 120 mm/min	Analysis of defect of joint	Defects occurred with high WS, high turbulence and insufficient plastic deformation. Although a truncated tool reduces defects compared to a cylindrical tool, 100 mm/min and 1500 rpm are the best choices.	[11]
AZ31 (B)	Cylindrical SKD61 tool steel (H13 steel)	RS—1000, 500 rpm WS—200, 300, 700 mm/min	TS, microstructure, texture, grain size	Tensile strength increased in DFSW due to the two tools. Lower tool joints show different mechanical properties; different speeds between upper and lower joints is necessary.	[21]
AZ31B (B)	Cylindrical tool	RS—1200 rpm WS—50 mm/min	TS, microstructure, texture, grain size, Hn	Stir zone has fine structure, hardness of SZ was more than TMAZ, RS had more deformation	[22]
AZ91 (C)	Thread cylinder pin Tapper cylinder pin Straight cylinder pin HCHCrDZ	RS—710, 1000, 1400 rpm WS—28, 40, 56 mm/min	TS, microstructure	Heat generator was helpful in proper welding, TSC of shoulder dia. 18 mm, RS 710 rpm, WS 28 mm/min suitable for welding with high tensile strength	[13]
6061-T4Al (A) and AZ31BMg (B)	Frustum-shaped right-ended thread	RS—400–800 rpm WS—50 mm/min	Microstructure, TS, Hn	Material flow increased at 145 °C. Mechanical interlocking increased.	[23]
6061-T4Al (A) and AZ31BMg (B)	Concentric circle	RS—1000 rpm WS—60 mm/min	Microstructure, TS, Hn, fractography	The best result was obtained at 1000 rpm, 60 mm/min and ultrasonic power 1400 W.	[24]
AZ31 (B) and AZ91 (C)	H13 tool steel, tapered 3 to 1	RS—1800, 1600, 1400 rpm, WS—100, 50, 25	Microstructure, TS, Hn	FSW avoids hot cracks, nugget zone fine grain, and is used to join dissimilar MgAl alloys	[25]

**Table 2:** Potentiodynamic corrosion tests of AA6061, AZ31B, and AZ91.

Workpiece Material	Operation	Solution Used for Potentiodynamic Polarisation	Ref.
AA6061 T6 (A)	FSW	Immersion tests in sodium chloride + hydrogen peroxide solution.	[26]
AA6061 (A) alloy and AZ31B (B)	FSW	The polarization tests were carried out in a corrosion cell containing 500 mL of NaCl solution.	[27]
AA6061 (A)	FSW	The test solution was also 3.5% NaCl. Potentiodynamic polarization curves were obtained in the potential range from −2.5 V to 2 V with a scan rate of 1.0 mV s^−1^.	[28]
AZ31 (B) and AZ91 (C)	Only polarization test, no FSW	Electrochemical impedance spectroscopy (EIS) and potentiodynamic polarization (PDP) techniques were employed to compare the performance of the alloys in these different aggressive electrolytes.	[29]
AZ31 (B) and AZ91 (AZ31B and AZ91)	Only polarization test, no FSW	Potentiodynamic polarization tests in 0.001 M NaCl	[30]
AZ31 (B) and AZ91 (AZ31B and AZ91)	Only polarization test, no FSW	studied in Hank’s solution, Dulbecco’s modified Eagle’s medium (DMEM) and serum-containing medium (DMEM adding 10% fetal bovine serum (DMEM + FBS)) over a 7-day immersion period	[31]
AZ31 (B) and AZ91 (C)	Only polarization test, no FSW	------	[32]

**Table 3:** Content of Mg and Al alloys (in mass fraction, %).

Material	Symbol	Al	Mn	Zn	Si	Fe	Cu	Ni	Others	Mg
Al6061-6	A	Bal	0.15	0.25	0.40–0.80	0.70	0.15–0.40	–	Cr 0.04–0.35 Ti 0.15	0.80–1.20
AZ31B	B	2.5–3.5	0.20–0.00	0.60–0.40	0.10	0.005	0.04	0.005	0.30	Bal.
AZ91D	C	8.3–9.7	0.15–0.50	0.35–1.00	0.10	0.005	0.03	0.002	0.02	Bal.

**Table 4:** Observations of hardness, impact energy, corrosion, and tensile properties.

FSW Joints	Observations	AA6061 and AZ31B	AZ31 and AA6061	AZ31B and AZ91	AZ91 and AZ31B	AA6061 and AZ91	AZ91 and AA6061
Tensile Strength (MPa)	1	146.35	139.53	185.37	191.62	175.91	177.321
	2	145.09	135.702	189.54	194.56	178.41	179.406
	3	139.54	135.552	188.4	190.99	176.47	177.648
	Avg.	143.66	136.928	187.77	192.39	176.93	178.125
Hardness (Hv)	1	113.57	83.41	104.29	100.73	99.7	99.51
	2	116.21	89.26	107.51	95.81	103	102.31
	3	112.22	84.34	103.2	99.47	103.3	103.19
	Avg.	114	85.67	105	98.67	102	101.67
Impact energy (Joules)	1	8.1	7.6	5.3	5.4	7.1	7.8
	2	8.5	8.5	5.5	5.6	7.9	8.5
	3	8.3	7.9	5.7	5.8	7.5	7.7
	Avg.	8.3	8	5.5	5.6	7.5	8
Corrosion Rate (mm/a mm year)	1	7.01	9.46	10.61	17.9	8.21	11.03
	2	7.03	9.53	10.71	18.4	8.29	11.06
	3	7.05	9.54	10.76	18.3	8.28	11.063
	Avg.	7.03,	9.51	10.69	18.2	8.26	11.051
Elongation (%)	1	2.31	3.1	6.23	6.4	9.3	8.06
	2	2.4	2.52	7.17	7.6	8.2	7.34
	3	3.21	2.3	7.6	7	8.3	8
	Avg.	2.64	2.64	7	7	8.6	7.8
Yield Strength (MPa)	1	114.9	117.24	106.4	106.86	134.21	123.36
	2	113.5	118.14	107.3	107.31	135.065	125.103
	3	114.8	117.3	106.13	106.539	133.85	124.287
	Avg.	114.4	117.56	106.61	106.903	134.375	124.25

**Table 5:** Cost calculations for FSW.

Workpiece	Cost of 1 Piece	TC_material_ (4 Piece)	CNC Cutting Per Piece	TC_cnc_ (4 Piece)	TC_welding_ (2 Piece)
A	23.79	95.16	83.33	333.33	616.66
B	135	540	83.33	333.33	616.66
C	157.5	630	83.33	333.33	616.66

## Data Availability

Not applicable.

## Nomenclature

A	AA6061
AS	Advancing side
Al	Aluminum
*b_a_*	Anodic Tafel slope
B	AZ31B
C	AZ91
BM	Base metal
*b_c_*	Cathodic Tafel slope
c	Chloride ion concentration
Cor	Corrosion
η	Corrosion rate
*J_corr_*	Current density
ρ	Density
K	Electrochemical equivalent
Elo	Elongation
t	Exposure time
FSW	Friction stir welding
Hn	Hardness
HAZ	Heat-affected zone
IS	Impact energy
Mg	Magnesium
F	Metal factor
MMC	Metal matrix composite
P	pH value
*R_p_*	Polarization resistance
RS	Retreating side
R_s	Rotational speed
SEM	Scanning electron microscopy
SZ	Stir zone
TS	Tensile strength
TMAZ	Thermomechanical heat-affected zone
TTA	Tool-tilt angle
TIG	Tungsten inert gas welding
WS	Welding speed
YS	Yield strength

## Appendix A

**Table A1:** Correlations between mechanical properties of FSW butt joint.

		Hardness	Tensile Strength	Impact Energy	Corrosion	Elongation	Yield Strength
Hardness	P_CoR	1	0.183	−0.003	−0.272	0.11	−0.078
	Sig. (2-tailed)	-	0.729	0.996	0.603	0.836	0.883
Tensile strength	P_CoR	0.183	1	−0.767	0.634	0.888 *	−0.145
	Sig. (2-tailed)	0.729	-	0.075	0.177	0.018	0.785
Impact energy	P_CoR	−0.003	−0.767	1	−0.7	−0.449	0.629
	Sig. (2-tailed)	0.996	0.075	−	0.122	0.371	0.181
Corrosion	P_CoR	−0.272	0.634	−0.7	1	0.351	−0.509
	Sig. (2-tailed)	0.603	0.177	0.122	-	0.495	0.303
Elongation	P_CoR	0.11	0.888 *	−0.449	0.351	1	0.318
	Sig. (2-tailed)	0.836	0.018	0.371	0.495	-	0.539
Yield strength	P_CoR	−0.078	−0.145	0.629	−0.509	0.318	1
	Sig. (2-tailed)	0.883	0.785	0.181	0.303	0.539	-

The “*” represents” Tensile Strength has very high degree positive significant correlation with elongation”. The “*p* value” represents “probability significance”.

**Table A2:** Model summary describing the strength of relationship among variables.

Model	Predictors (Constant):	Dependent VariAA6061 and AZ31Ble	R	R Square	Adjusted R Square	Std. Error of the Estimate	R Square Change	F Change	df1	df2	Sig. F Change	Durbin-Watson
Model 1	Yield strength, elongation	Tensile strength	0.996 a	0.993	0.988	2.58279	0.993	202.055	2	3	0.001	2.166
Model 2	Tensile strength	Hardness	0.183 a	0.033	−0.208	10.15492	0.033	0.138	1	4	0.729	2.629

The “a” represents “Relationship has been built between tensile strength, yield strength, hardness and elongation in Model 1, Model 2”.

**Table A3:** ANOVA of statistical differences among variables.

Model	Predictors (Constant):	Dependent Variable		Sum of Squares	df	Mean Square	F	Sig.
Model 1	Yield strength, elongation	Tensile Strength	Regression	2695.73	2	1347.87	202.055	0.001 b
			Residual	20.012	3	6.671		
			Total	2715.74	5			
Model 2	Tensile strength	Hardness	Regression	14.227	1	14.227	0.138	0.729 b
			Residual	412.489	4	103.122		
			Total	426.717	5			

The “b” represents “significant statistical difference between tensile strength, yield strength, hardness and elongation in Model 1 and Model 2”.

**Table A4:** Linear relationship between tensile strength with elongation and yield strength.

Model 1	**Variables**		**Unstandardized Coefficients**		**Standardized Coefficients**	**t**	**Sig.**
	**Dependent Variable**	**Independent Variables**	**B**	**Std. Error**	**Beta**		
	Tensile strength	(Constant)	236.222	12.82		18.427	0
		Elongation	9.218	0.463	1.04	19.89	0
		Yield strength	−1.037	0.114	−0.476	−9.096	0.003

**Table A5:** Linear relationship between hardness and tensile strength.

Model 2	**Variables**		**Unstandardized Coefficients**		**Standardized Coefficients**	**t**	**Sig.**
	**Dependent Variable**	**Independent Variable**	**Independent Variables**	**Std. Error**	**Beta**		
	Hardness	(Constant)	88.914	33.250		2.674	0.056
		Tensile strength	0.072	0.195	0.183	0.371	0.729

## References

[1] Luo A., Sachdev A. (2010). Microstructure and Mechanical Properties of Magnesium-Aluminum-Manganese Cast Alloys. Int. J. Met..

[2] Yang J., Oliveira J.P., Li Y., Tan C., Gao C., Zhao Y., Yu Z. (2022). Laser techniques for dissimilar joining of aluminum alloys to steels: A critical review. J. Mater. Process. Technol..

[3] Flenady V., Koopmans L., Middleton P., Frøen J.F., Smith G.C., Gibbons K., Coory M., Gordon A., Ellwood D., McIntyre H.D. (2011). Major risk factors for stillbirth in high-income countries: A systematic review and meta-analysis. Lancet.

[4] Zeng R.-C., Dietzel W., Zettler R., Gan W.-M., Sun X.-X. (2014). Microstructural evolution and delayed hydride cracking of FSW-AZ31 magnesium alloy during SSRT. Trans. Nonferrous Met. Soc. China.

[5] Cao X., Jahazi M. (2009). Effect of Welding Speed on the Quality of Friction Stir Welded Butt Joints of a Magnesium Alloy. Mater. Des..

[6] Wang K.-S., Wu J.-L., Wang W., Zhou L.-H., Lin Z.-X., Kong L. (2012). Underwater friction stir welding of ultrafine grained 2017 aluminum alloy. J. Cent. South Univ..

[7] Fantetti N., Pekguleryuz M.O., Avedesian M.M., Sahoo M., Pinfold P. (1991). Magnesium plaster cast prototypes versus diecastings—A comparative evalution of properties. Extraction, Refining, and Fabrication of Light Metals.

[8] Cao X., Jahazi M., Immarigeon J.P., Wallace W. (2006). A review of laser welding techniques for magnesium alloys. J. Mater. Process. Technol..

[9] Ke W.C., Oliveira J.P., Ao S.S., Teshome F.B., Chen L., Peng B., Zeng Z. (2022). Thermal process and material flow during dissimilar double-sided friction stir spot welding of AZ31/ZK60 magnesium alloys. J. Mater. Res. Technol..

[10] Dargusch M.S., Dunlop G.L., Bowles A.L., Pettersen K., Bakke P. (2004). The effect of silicon content on the microstructure and creep behavior in die-cast magnesium AS alloys. Metall. Mater. Trans. A.

[11] Gulati P., Shukla D.K., Gupta A. (2017). Defect formation analysis of Friction Stir welded Magnesium AZ31B alloy. Mater. Today Proc..

[12] Lambrakos S.G. (2018). Parametric Modeling of AZ31-Mg Alloy Friction Stir Weld Temperature Histories. J. Mater. Eng. Perform..

[13] Patel N., Bhatt K.D., Mehta V. (2016). Influence of Tool Pin Profile and Welding Parameter on Tensile Strength of Magnesium Alloy AZ91 During FSW. Procedia Technol..

[14] Malarvizhi S., Balasubramanian V. (2012). Influences of tool shoulder diameter to plate thickness ratio (D/T) on stir zone formation and tensile properties of friction stir welded dissimilar joints of AA6061 aluminum–AZ31B magnesium alloys. Mater. Des..

[15] Bandi A., Bakshi S.R. (2020). Effect of Pin Length and Rotation Speed on the Microstructure and Mechanical Properties of Friction Stir Welded Lap Joints of AZ31B-H24 Mg Alloy and AA6061-T6 Al Alloy. Metall. Mater. Trans. A Phys. Metall. Mater. Sci..

[16] Martinsen K., Hu S.J., Carlson B.E. (2015). Joining of dissimilar materials. CIRP Ann..

[17] Sunil B.R., Ganesh K.V., Pavan P., Vadapalli G., Swarnalatha C., Swapna P., Bindukumar P., Pradeep Kumar Reddy G. (2016). Effect of aluminum content on machining characteristics of AZ31 and AZ91 magnesium alloys during drilling. J. Magnes. Alloy.

[18] El Rayes M.M., Soliman M.S., Abbas A.T., Pimenov D.Y., Erdakov I.N., Abdel-mawla M.M. (2019). Effect of Feed Rate in FSW on the Mechanical and Microstructural Properties of AA5754 Joints. Adv. Mater. Sci. Eng..

[19] Sankar B.R., Umamaheswarrao P. (2017). Modelling and optimization of friction stir welding on AA6061 Alloy. Mater. Today Proc..

[20] Huang Y., Meng X., Wang Y., Xie Y., Zhou L. (2018). Joining of aluminum alloy and polymer via friction stir lap welding. J. Mater. Process. Technol..

[21] Tripathi A., Murty S.V.S.N., Narayanan P.R. (2017). Microstructure and texture evolution in AZ31 magnesium alloy during caliber rolling at different temperatures. J. Magnes. Alloy.

[22] Dorbane A., Ayoub G., Mansoor B., Hamade R.F., Kridli G., Shabadi R., Imad A. (2016). Microstructural observations and tensile fracture behavior of FSW twin roll cast AZ31 Mg sheets. Mater. Sci. Eng. A.

[23] Lv X., Wu C., Yang C., Padhy G.K. (2018). Weld microstructure and mechanical properties in ultrasonic enhanced friction stir welding of Al alloy to Mg alloy. J. Mater. Process. Technol..

[24] Liu Z., Meng X., Ji S., Li Z., Wang L. (2018). Improving tensile properties of Al/Mg joint by smashing intermetallic compounds via ultrasonic-assisted stationary shoulder friction stir welding. J. Manuf. Process..

[25] Ratna Sunil B., Pradeep Kumar Reddy G., Mounika A.S.N., Navya Sree P., Rama Pinneswari P., Ambica I., Ajay Babu R., Amarnadh P. (2015). Joining of AZ31 and AZ91 Mg alloys by friction stir welding. J. Magnes. Alloy.

[26] Gharavi F., Matori K.A., Yunus R., Othman N.K., Fadaeifard F. (2016). Corrosion evaluation of friction stir welded lap joints of AA6061-T6 aluminum alloy. Trans. Nonferrous Met. Soc. China.

[27] Jayaraj R.K., Malarvizhi S., Balasubramanian V. (2017). Electrochemical corrosion behaviour of stir zone of friction stir welded dissimilar joints of AA6061 aluminium–AZ31B magnesium alloys. Trans. Nonferrous Met. Soc. China.

[28] El-Deeb M.S.S., Khorshed L.A., Abdallah S.A., Gaafer A.M., Mahmoud T.S. (2019). Effect of friction stir welding process parameters and post-weld heat treatment on the corrosion behaviour of AA6061-O aluminum alloys. Egypt. J. Chem..

[29] Mena-Morcillo E., Veleva L. (2020). Degradation of AZ31 and AZ91 magnesium alloys in different physiological media: Effect of surface layer stability on electrochemical behaviour. J. Magnes. Alloy.

[30] Fritzsch K., Zenker R., Buchwalder A. (2015). Improved Surface Properties of AZ31 and AZ91 Mg alloys Due to Electron Beam Liquid Phase Surface Treatment. Proc. Mater. Today Proc..

[31] Gu X.N., Zheng Y.F., Chen L.J. (2009). Influence of artificial biological fluid composition on the biocorrosion of potential orthopedic Mg-Ca, AZ31, AZ91 alloys. Biomed. Mater..

[32] Mahallawy N.E., Harhash M. (2013). Recent studies on coating of some magnesium alloys; Anodizing, Electroless coating and hot press cladding. Key Eng. Mater..

[33] Aval H.J., Loureiro A. (2019). Effect of reverse dual rotation process on properties of friction stir welding of AA7075 to AISI304. Trans. Nonferrous Met. Soc. China.

[34] Zhao J., Wu C., Su H. (2021). Acoustic effect on the tensile properties and metallurgical structures of dissimilar friction stir welding joints of Al/Mg alloys. J. Manuf. Process..

[35] Verma S., Kumar V., Kumar R., Sidhu R.S. (2022). Exploring the application domain of friction stir welding in aluminum and other alloys. Mater. Today Proc..

[36] Elangovan K., Balasubramanian V. (2008). Influences of tool pin profile and tool shoulder diameter on the formation of friction stir processing zone in AA6061 aluminium alloy. Mater. Des..

[37] Darras B., Khraisheh M., Abu-Farha F., Omar M. (2007). Friction stir processing of commercial AZ31 magnesium alloy. J. Mater. Process. Technol..

[38] Nelson T., Steel R., Arbegast W. (2003). In situ thermal studies and post-weld mechanical properties of friction stir welds in age hardenable aluminium alloys. Sci. Technol. Weld. Join..

[39] Upadhyay P., Reynolds A.P. (2010). Effects of thermal boundary conditions in friction stir welded AA7050-T7 sheets. Mater. Sci. Eng. A.

[40] Cabibbo M., McQueen H., Evangelista E., Spigarelli S., Di Paola M., Falchero A. (2007). Microstructure and mechanical property studies of AA6056 friction stir welded plate. Mater. Sci. Eng. A.

[41] Safeen W., Hussain S., Wasim A., Jahanzaib M., Aziz H., Abdalla H. (2016). Predicting the tensile strength, impact toughness, and hardness of friction stir-welded AA6061-T6 using response surface methodology. Int. J. Adv. Manuf. Technol..

[42] Bilgin M., Karabulut S., Özdemir A. Study on the Mechanical Properties of Dissimilar Friction Stir Welding of AA 7075 T6 and AZ31B Alloys. Proceedings of the 2018 9th International Conference on Mechanical and Aerospace Engineering, (ICMAE 2018).

[43] Chauhan A., Kumar S. (2018). Effect of friction stir welding parameters on impact strength of the AZ31 magnesium alloy joints. Int. J. Mech. Prod. Eng. Res. Dev..

[44] Liu Z., Xin R., Liu D., Shu X., Liu Q. (2016). Textural variation in triple junction region of friction stir welded Mg alloys and its influence on twinning and fracture. Mater. Sci. Eng. A.

[45] Liu D., Xin R., Sun L., Zhou Z., Liu Q. (2013). Influence of sampling design on tensile properties and fracture behavior of friction stir welded magnesium alloys. Mater. Sci. Eng. A.

[46] Afrin N., Chen D., Cao X., Jahazi M. (2008). Microstructure and tensile properties of friction stir welded AZ31B magnesium alloy. Mater. Sci. Eng. A.

[47] Xunhong W., Kuaishe W. (2006). Microstructure and properties of friction stir butt-welded AZ31 magnesium alloy. Mater. Sci. Eng. A.

[48] Chowdhury S.M., Chen D.L., Bhole S.D., Cao X. (2010). Tensile properties of a friction stir welded magnesium alloy: Effect of pin tool thread orientation and weld pitch. Mater. Sci. Eng. A.

[49] Singh S., Kumar R., Kumar R., Chohan J.S., Ranjan N., Kumar R. (2022). Aluminum metal composites primed by fused deposition modeling-assisted investment casting: Hardness, surface, wear, and dimensional properties. Proc. Inst. Mech. Eng. Part L J. Mat. Des. Appl..

[50] Kumar R., Channi H.K. (2022). A PV-Biomass off-grid hybrid renewable energy system (HRES) for rural electrification: Design, optimization and techno-economic-environmental analysis. J. Clean. Prod..

[51] Goyal K.K., Sharma N., Gupta R.D., Singh G., Rani D., Banga H.K., Kumar R., Pimenov D.Y., Giasin K. (2022). A Soft Computing-Based Analysis of Cutting Rate and Recast Layer Thickness for AZ31 Alloy on WEDM Using RSM-MOPSO. Materials.

[52] Sidhu A.S., Singh S., Kumar R., Pimenov D.Y., Giasin K. (2021). Prioritizing Energy-Intensive Machining Operations and Gauging the Influence of Electric Parameters: An Industrial Case Study. Energies.

[53] Kumar R., Kaur S. Multi Attribute Decision Making Approach to Select Microwave Oven with TOPSIS Method. Proceedings of the 7th International Conference on Advancements in Engineering and Technology (ICAET-2019).

